# An Interspecies Molecular and Functional Study of Organic Cation Transporters at the Blood-Brain Barrier: From Rodents to Humans

**DOI:** 10.3390/pharmaceutics12040308

**Published:** 2020-03-28

**Authors:** Catarina Chaves, Federica Campanelli, Hélène Chapy, David Gomez-Zepeda, Fabienne Glacial, Maria Smirnova, Meryam Taghi, Johan Pallud, Nicolas Perrière, Xavier Declèves, Marie-Claude Menet, Salvatore Cisternino

**Affiliations:** 1 Inserm, U1144, Optimisation Thérapeutique en Neuropsychopharmacologie, 75006 Paris, France; catarinachaves1987@gmail.com (C.C.); federica.cam@hotmail.it (F.C.); helene.chapy@gmail.com (H.C.); maria.smirnova@inserm.fr (M.S.); meryam.taghi@parisdescartes.fr (M.T.); xavier.decleves@parisdescartes.fr (X.D.); marie-claude.menet@parisdescartes.fr (M.-C.M.); 2 Faculté de pharmacie, Université de Paris, UMR-S 1144, 4, Avenue de l’Observatoire, 75006 Paris, France; 3 BrainPlotting SAS, Institut du Cerveau et de la Moelle épinière, 75013 Paris, France; fabienne.glacial@brainplotting.com (F.G.); nicolas.perriere@brainplotting.com (N.P.); 4 Department of Neurosurgery, Sainte Anne Hospital, 75014 Paris, France; j.pallud@chu-st-anne.fr; 5 Inserm, U894, IMA-Brain, Centre de Psychiatrie et Neurosciences, 75013 Paris, France; 6 Assistance Publique-Hôpitaux de Paris, AP-HP, Hôpital Universitaire Cochin, Biologie du médicament et toxicologie, 75006 Paris, France; 7 Assistance Publique-Hôpitaux de Paris, AP-HP, Hôpital Universitaire Cochin, Hormonologie adulte, 75006 Paris, France; 8 Assistance Publique-Hôpitaux de Paris, AP-HP, Hôpital Universitaire Necker-Enfants Malades, Service de pharmacie, 75015 Paris, France

**Keywords:** biological transport, blood-brain barrier, drug delivery, neurotoxicity, organic cations, solute carriers

## Abstract

Organic cation transporters (OCTs) participate in the handling of compounds in kidneys and at the synaptic cleft. Their role at the blood-brain barrier (BBB) in brain drug delivery is still unclear. The presence of OCT1,2,3 (SLC22A1-3) in mouse, rat and human isolated brain microvessels was investigated by either qRT-PCR, quantitative proteomics and/or functional studies. BBB transport of the prototypical substrate [^3^H]-1-methyl-4-phenylpyridinium ([^3^H]-MPP^+^) was measured by in situ brain perfusion in six mouse strains and in Sprague Dawley rats, in primary human brain microvascular endothelial cells seeded on inserts, in the presence or absence of OCTs and a MATE1 (SLC49A1) inhibitor. The results show negligible OCT1 (SLC22A1) and OCT2 (SLC22A2) expression in either mice, rat or human brain microvessels, while OCT3 expression was identified in rat microvessels by qRT-PCR. The in vitro human cellular uptake of [^3^H]-MPP^+^ was not modified by OCTs/MATE-inhibitor. Brain transport of [^3^H]-MPP^+^ remains unchanged between 2- and 6-month old mice, and no alteration was observed in mice and rats with inhibitors. In conclusion, the evidenced lack of expression and/or functional OCTs and MATE at the BBB allows the maintenance of the brain homeostasis and function as it prevents an easy access of their neurotoxicant substrates to the brain parenchyma.

## 1. Introduction

Neurons and glial cells forming the brain parenchyma are surrounded by the interstitial fluid (ISF). Within the brain parenchyma, endothelial or epithelial cells held together by tight junctions are found constituting barriers to the paracellular route and controlling the transcellular molecular exchanges between the blood and the brain fluid spaces. Indeed, the blood-brain barrier (BBB), formed by the cerebrovascular endothelial cells, plays a critical role in regulating the molecular trafficking between the blood and the ISF. The presence/absence of systemic endo or xenobiotic in the ISF is also modulated by biochemical features present at the BBB (e.g., transporter proteins). Compounds may cross the BBB and reach the brain parenchyma/ISF through a transcellular route, namely passive and/or carrier-mediated transport. The brain drug delivery is limited by ATP-binding cassette (ABC) efflux transporters like P-glycoprotein (P-gp; ABCB1) present at the luminal membrane of the brain microvessel endothelial cells [1]. On the other hand, the solute carrier (SLC) transport superfamily can possibly mediate the import of substrates into the brain endothelium, and from there into the central nervous system (CNS)/ISF, which may represent further hope in the development of CNS-active drugs and new drug delivery strategies, but also the vulnerability of the BBB to the entrance of neurotoxicants into the CNS. The concentration of compounds within the ISF, and consequently its composition, is also affected by neurons and glial cells due to the expression of neurotransmitter “uptake” transporters known as DAT, NET, SERT (SLC6A family) and OCT2-3 (SLC22A2-3), potassium channels (Kir 4.1) and glutamate transporters (SLC1A).

Several SLCs have been identified to be present at the BBB and to contribute to the carrier-mediated transport into the CNS, such as GLUT1 (SLC2A1) [2,3], LAT1 (SLC7A5) [4,5] or CAT1 (SLC7A1) [6]. The expression of organic cation transporters (OCTs) and other SLCs is still a matter of debate, which needs further elucidation. OCTs are mainly implicated in the detoxification of endogenous and xenobiotic cationic compounds, due to its substrate specificity and localization – OCT1 (SLC22A1) primarily expressed in hepatocytes, and OCT2 (SLC22A2) primarily expressed in the kidney. Therefore, an OCT1/OCT2 expression at the luminal BBB could be inconsistent with its function, as it would render any substrate of OCTs – many of them neurotoxic compounds, such as paraquat, 1-Methyl-4-Phenylpyridinium (MPP^+^), and several endobiotics like dopamine and uremic toxins [7]—an easy access to the CNS and compromise its function and homeostasis.

Several studies have demonstrated that neither OCT1 (SLC22A1) nor OCT2 (SLC22A2) are expressed in FVB and Swiss mice strains and in human isolated brain microvessels [8,9,10,11]. Controversially, both OCT1 and/or OCT2 were claimed to be expressed at the luminal side of human, mice and rat brain endothelial cells, and to account for more than 90% of 1-methyl-4-phenyl-1,2,3,6-tetrahydropyridine (MPTP) brain uptake [12]. Interestingly, a report also suggests that Oct1 and Oct2 expression at the BBB are dependent on the mouse strain, evidencing an age-declining Oct1-2 expression in 2- versus 6-months old C57BL/6 but not in Swiss mice, and thus a higher neuro-sensitivity to their substrates at a young age, while both strains lack Oct3 [9]. These results have led to speculation that the variability of the neurotoxic doses of MPP^+^/MPTP observed between many rodent strains might depend on the expression of Oct1/2 at their BBB [9].

In this study, we aimed at clarifying the current controversy regarding the eventual strain- and age-dependent presence and function of OCTs and MATE1 at the BBB. For this matter, we analyzed the expression, at mRNA and protein level, and/or function of OCTs and MATE1 in brain microvessels from mice of different strains and age (2- and 6-months old), but also in rat and human brain microvessels. For the functional studies, we measured the transport of the cationic metabolite of MPTP, [^3^H]-MPP^+^, a known substrate of transporters such as OCTs and MATE at the BBB of FVB, NMRI, DBA/1J, BALB/c, C3H/HeNR and C57BL/6 mice and of Sprague Dawley rats by in situ brain perfusion, and in human primary brain endothelial cells cultured using a transwell system. Our present results further support very low or negligible mRNA levels of Octs in rodents’ brain microvessels, and an evident lack of expression of the protein level in humans. The general lack of carrier-mediated [^3^H]-MPP^+^ BBB transport, regardless of the species, further supports a lack of OCTs and MATE BBB function.

## 2. Materials and Methods

### 2.1. Chemicals and Reagents

[^14^C]-Sucrose (580–600 mCi/mmol), [^3^H]-L-phenylalanine (128 Ci/mmol) and [^3^H]-MPP^+^ (70–84 Ci/mmol) were purchased from Perkin Elmer (Paris, France). Protease Inhibitor Cocktail Complete Mini^®^ was purchased from Roche (Basel, Switzerland). Protease MAX surfactant, mass spectrometry grade rLys-C and sequencing grade modified trypsin were acquired from Promega (Charbonnières-les-Bains, France). RIPA buffer was prepared employing analytical grade reagents from Sigma Aldrich (Saint Quentin Fallavier, France): 50 mmol L^−1^ Tris (pH 8.0), 150 mmol L^−1^ NaCl, 1% (V/V) Triton X-100, 0.1% (V/V) sodium dodecyl sulfate (SDS) and 0.5% (W/V) sodium deoxycholate in high purity water. Standard peptides for protein quantification were provided by Bertin Pharma (Orleans, France). Tetraethylammonium (TEA) was purchased from Sigma Aldrich. All other chemicals were of analytical grade.

### 2.2. Animals

Male FVB, C57BL/6, NMRI, DBA/1J, BALB/c, C3H/HeNR (2 months old) mice and Sprague Dawley rats (2 months old) were obtained from Janvier (Genest, France). In some functional studies, 6 months old mice from C57BL/6, DBA, NMRI and FVB strains were used. The mice and rats were housed in a controlled environment (19 ± 2 °C, 55 ± 10% relative humidity) with a 12-h light/dark cycle, and access to food and water *ad libitum*. All studies involving animals are reported in accordance with ARRIVE guidelines for reporting experiments involving animals and complied with the ethical rules of the European directive (210/63/EU) for experimentation with laboratory animals; they were approved by the ethics review committee of Paris Descartes University (approval n°12-183/12-2012).

### 2.3. Isolation of Rat, Mouse and Human Microvessels

#### 2.3.1. Isolation of Rodent Brain Microvessels:

Rat and mouse brain cortex vessels were isolated according to [13], minimizing contamination from astrocyte and neuron mRNA. Rats and mice were euthanized by CO_2_ inhalation. Animals were decapitated and brains were immediately removed and placed in ice-cold Hank’s buffered salt solution (HBSS), supplemented with HEPES at 10 mmol L^−1^. Isolated brain cortices were minced in HBSS, homogenized in a Potter Thomas homogenizer (0.25 mm clearance), and the resulting homogenates were centrifuged at 2000 *g* for 10 min at 4 °C. Each pellet was suspended in 17.5% dextran (64–76 kDa, Sigma Aldrich) and centrifuged at 4400 *g* for 15 min at 4 °C. The resulting pellets were suspended in HBSS containing 1% bovine serum albumin (BSA) and passed through a 100 µm nylon mesh. The filtrate was then passed through a 20 µm nylon mesh, where microvessels (mainly 4–6 µm) were retained and immediately collected. All steps were carried out at 4 °C.

#### 2.3.2. Isolation of Human Brain Microvessels:

All human samples were obtained from BrainPlotting (iPEPS, Institut du Cerveau et de la Moelle épinière, Hôpital Universitaire de la Pitié-Salpêtrière, Paris, France), and harvested under the authorization number (CODECOH DC-2014-2229) in partnership with Sainte-Anne Hospital, Paris (neurosurgeon Dr. Johan Pallud). Patients gave their written informed consent. Human brain capillaries were isolated from temporal cortex resections obtained from patients during tumor scheduled resection surgery (8 male, 5 females; age 31–73 years), diagnosed with grade II, III or IV glioma. Enzymatic isolation of human brain capillaries was adapted from previously described protocols in the rat [14,15]. Briefly, human brain samples were cleaned from meninges and excess of blood. Tissues were digested using an enzymatic mix and capillaries retained on a 10 µM mesh. A total of 13 brain microvessel samples were obtained, including 9 samples from pathological glioma tissue, two from peritumoral and two from non-pathological tissues. The human microvessels obtained were subsequently used for protein extraction, or cultured to originate primary human brain microvascular endothelial cells (BMVECs).

### 2.4. Human BMVECs and BBB Permeability Validation

#### 2.4.1. Primary Human Brain Microvascular Endothelial Cell Culture (BMVECs):

BMVECs were obtained from isolated human brain microvessels and cultured in petri dishes in the presence of puromycin (P0), to avoid contamination from other cell types. BMVECs were later used in astrocyte-conditioned culture media for protein extraction, or for the establishment of a human BBB model to conduct permeability assays.

#### 2.4.2. Phenylalanine and MPP^+^ Permeability Assays:

After BMVECs amplification, cells were seeded on inserts (Transwell, Corning, ThermoFisher, Les Ulis, France). Cell monolayer integrity and paracellular permeability to ions and hydrophilic molecules were assessed by measuring the transendothelial electrical resistance (TEER) and the permeability coefficient of [^14^C]-sucrose, respectively, as previously described [15]. Permeability studies using radiolabeled [^3^H]-L-Phenylalanine (~4 nM) or [^3^H]-MPP^+^ (~6 nM) were performed on BMVECs once the establishment of a good BBB model was verified. To further confirm the tightness of the cell monolayer in each experiment, [^14^C]-sucrose was systematically added to the permeability buffer, together with the [^3^H]-labeled molecule of interest (apical or basolateral chamber). ^3^H and ^14^C in the apical/basolateral samples were measured by dual-label counting using a TriCarb liquid scintillation radiolabeled counter (Perkin Elmer).

### 2.5. Extraction and Dosage of Total RNA from Rat and Mice Brain Microvessels

Total RNA from rat and mice brain microvessels were obtained through homogenization and lysis using TissueLyser system (Qiagen, Courtaboeuf, France) and subsequent extraction using the RNeasyQiagen Micro kit, according to the manufacturer’s instructions (Qiagen). Concentration and purity of the obtained RNA samples were assessed spectrophotometrically using a Nanodrop spectrophotometer (NanoDrop Technologies, Wilmington, DE, USA).

### 2.6. Reverse Transcription (RT) and Quantitative Real-Time PCR (qRT-PCR)

#### 2.6.1. cDNA Synthesis:

Total RNA samples were reverse transcribed into cDNA in a final volume of 20–50 µL. For each sample, RNAse-free water containing 100–1000 ng of total RNA was mixed with the RT reaction mixture (1X Buffer, 10 mM DTT, 500 µM dNTP, 1.5 µM Random Hexanucleotides primers, 20 U µL^−1^ RNase OUT, 100 U µL^−1^ Superscript II RNase H-Reverse Transcriptase in RNase-free water). All reagents were from Invitrogen™ (ThermoFisher, Les Ulis, France), with the exception of random hexanucleotides primers, which were from Roche (Roche Diagnostics, Meylan, France). For negative controls (RT^−^), the reverse transcriptase was replaced by RNAse-free water in the RT mix. RT was performed on a T100™ Thermal Cycler (Bio-Rad Laboratories, Marnes-la-Coquette, France) according to the following program: 25 °C for 10 min, 42 °C for 30 min, 99 °C for 5 min. cDNA samples (cDNAs) were then stored at −80 °C until analysis by qRT-PCR.

#### 2.6.2. RT-PCR Theoretical Principles:

The transcript levels for each target gene were investigated by qRT-PCR. Relative expressions of target genes were determined for samples with a gene cycle threshold (CT) lower than 33. Target genes were considered to be unquantifiable when CT values were above 33. To ensure the validity of the results, the efficiency of polymerase chain reaction PCR (E) was checked, and it was greater than 95% for all the tested genes. The ΔΔCT method was used to compare the expression of a gene of interest in rat and mice brain microvessels, which were normalized to that of the housekeeping gene (*β-actin*). This comparison was possible because the expression of the *β-actin* gene in rat and mice brain microvessels was not statistically different in any of the tested samples.

#### 2.6.3. PCR Amplification:

Specific nucleotide primer sequences (see Table 1) were designed using OLIGO 7 software (Medprobe, Oslo, Norway) and synthesized by Eurogentec (Angers, France). Amplification was detected by SYBR^®^ Green fluorescence on an ABI PRISM^®^ 7900HT sequence detection system (Applied Biosystems, Courtaboeuf, France). Thermocycling was carried out in a final volume of 20 µL. The thermal cycling conditions were 2 min at 50 °C, then 10 min at 95 °C, followed by 40 amplification cycles at 95 °C for 15 s, at 60 or 65 °C for 45 s and at 95 °C for 15 s. qPCR melting curve analysis was performed to confirm the specificity and efficiency of selected primers.

### 2.7. Protein Extraction

BMVECs at P0 (*n* = 3) and P1 (*n* = 4) cultured cells were washed twice with Dulbecco’s phosphate-buffered saline (DPBS) buffer and collected by scraping. Microvessel pellets (*n* = 5) were also washed twice with DPBS before lysis. Cell and microvessel pellets were ressuspended in RIPA lysis buffer and protein was performed by sonication (BioRuptor, Diagenode, Belgium). Samples were subsequently centrifuged at 10,000 g 4 °C for 10 min. Supernatants were collected (post-nuclear fraction) and the total protein amount was determined using the MicroBCA^®^ kit (Thermo Scientific, Illkirch, France).

### 2.8. Protein Digestion

Protein samples were digested as previously reported [15]. Briefly, denatured and alkylated proteins were cleaned by precipitation, using a methanol-chloroform-water mixture. The protein pellet was resuspended using urea and Protease-Max detergent in Tris-HCl buffer (pH 8.5), and digested in tandem using Lys-C and Trypsin endoproteases (enzyme-protein mass ratio = 1:50 and 1:100, respectively). Stable isotope labeled (SIL) peptides were added after digestion for absolute quantification. Samples were dried using a centrifugal vacuum concentrator (Maxi-Dry Lyo, Heto Lab Equipment, Roskilde, Denmark), stored at −80 °C and solubilized just before analysis in an aqueous mixture containing 10% acetonitrile plus 0.1% formic acid.

### 2.9. Multiple Reaction Monitoring Quantification

Protein quantification was performed using the AQUA approach [16]. The sequences of peptides (sequences in Table 2) used for protein quantification have been previously described [11,17]. Peptides were synthetized in light and heavy (SIL) forms as standards. A calibration curve was prepared, including the light peptides covering a range from 0.125 to 62.5 fmol µL^−1^ and the heavy peptides in constant concentration (15 fmol µL^−1^) in a 10% acetonitrile, 0.1% formic acid aqueous solution. A QuaSAR plugin [18] was used to obtain the linear regressions, as well as the limits of detection (LOD) and the lower limits of quantification (LLOQ). The linear regression showed a R^2^ above 0.98 for all peptides (Table 2).

Targeted liquid chromatography with tandem mass spectrometry (LC-MS/MS) analyses were performed employing an ACQUITY UPLC H-Class^®^ System in line with a Waters Xevo^®^ TQ-S mass spectrometer (Waters, Manchester, UK). Peptides were injected into an ACQUITY UPLC BEH^®^ C18 column (Peptide BEH^®^ C18 Column, 300Å, 1.7 µm, 2.1 mm × 100 mm; Guyancourt, France) and eluted over a 24 min, gradient going from 100% of mobile phase A (water + 0.1% (V//V) formic acid) to 35% (V/V) of mobile phase B (acetonitrile + 0.1% (V//V) formic acid), with a flow rate of 0.3 mL min^−1^. Eluted molecules were ionized in positive electrospray, with ion spray capillary voltage at 2.80 kV, cone voltage at 35 V, drying gas flow-rate at 1000 L h^−1^ and a temperature of 650 °C. An analysis was performed in multiple reaction monitoring (MRM) mode using three to four transitions per peptide. The transitions used for quantification have been previously reported [19]. The collision energy was optimized as previously described [20]. Skyline [21] software (version 3.5.0.9319, MacCoss Lab Software, Seattle, WA, USA) was used for MRM method development, peak integration and peptide quantification using a linear regression without weighting.

### 2.10. Mice and Rat BBB Transport Studies by In situ Brain Perfusion

Transport of [^3^H]-MPP^+^ at the luminal BBB was measured by in situ brain perfusion in mice and rats [22,23,24]. We used the same in situ brain perfusion methodology as reported by André et al. [8], which made it possible to show the role of Oct1/2 at the level of the blood-retinal barrier and their absence at the BBB in Swiss mice using [^3^H]-MPP^+^ as a probe substrate. With this method, the vascular composition of the brain is totally substituted by an artificial fluid, whose constitution can be modified. Mice and rats were anesthetized with ketamine-xylazine mixture 140–8 mg kg^−1^ and 80-10 mg kg^−1^, respectively. A catheter was inserted into the right carotid artery after ligation of the appropriate vessels. Just before perfusion, the heart was cut. Perfusion started immediately at a constant flow rate of 2.5 mL min^−1^, and 10 mL min^−1^, in mouse and rat, respectively.

The perfusion fluid was Krebs-carbonate buffered physiological saline (mmol L^−1^): 128 NaCl, 24 NaHCO_3_, 4.2 KCl, 2.4 NaH_2_PO_4_, 1.5 CaCl_2_, 0.9 MgSO_4_, and 9 D-glucose, warmed to 37 °C and gassed with 95% O_2_/5% CO_2_ to bring the pH to 7.40. The fluid pH (pH_e_) was checked and adjusted with a digital pH-meter (± 0.05 pH-units) immediately before perfusion. Each animal was perfused with [^3^H]-MPP^+^ (~0.011 MBq mL^−1^; ~6 nM), and a vascular marker [^14^C]-sucrose (~0.0037 MBq mL^−1^), with or without tetraethylammonium (TEA; 30 mM). The perfusion was terminated by decapitating the animal after 120 s. The cerebral hemisphere was removed from the skull and dissected on a freezer pack. The tissues and aliquots of perfusion fluid were weighted, digested (Solvable^®^; Perkin Elmer) and mixed with Ultima-gold XR^®^ (Perkin Elmer). Dual-label counting was carried out in a Tri-Carb 2810TR (Perkin Elmer) to measure disintegrations per minute (dpm).

#### Apparent Initial Tissue Distribution Volume and Transport Parameters

Calculations were performed as previously described [8,22]. The brain “vascular” volume was estimated using the [^14^C]-sucrose distribution volume (V_v_; µL g^−1^) (Equation (1)):

(1) $$V_{v}=\frac{X^{*}}{C_{perf}^{*}}$$

where X* (dpm g^−1^) is the amount of [^14^C]-sucrose in the right brain hemisphere and C*_perf_ (dpm µL^−1^) is the [^14^C]-sucrose concentration in the perfusion fluid. The data for any animal whose V*_v_* was above the normal value [22] were excluded from the study.

The apparent tissue distribution volume (V_tissue_, µL g^−1^) was calculated as (Equation (2)):

(2) $$V_{tissue}=\frac{V_{tissue}}{C_{perf}}$$

where X_tissue_ (dpm g^−1^) is the calculated brain amount of [^3^H]-MPP^+^ and C_perf_ (dpm µL^−1^), its concentration in the perfusion fluid (Equation (3)):

(3) $$X_{tissue}=X_{tot}-V_{v}C_{perf}$$

where X_tot_ (dpm g^−1^) is the total quantity of [^3^H]-MPP^+^ measured in the sample tissue. The amount of [^3^H]-MPP in the vascular “[^14^C]-sucrose” space (V_v_. C_perf_) was calculated and subtracted from the total (X_tot_) (Equation (3)).

The initial transport rate, also called brain clearance, expressed as a K_in_ (µL s^−1^ g^−1^), was calculated from (Equation (4)):

(4) $$K_{in}=\frac{V_{tissue}}{T}$$

where T is the perfusion time (s).

Extraction E (%) is given by (Equation (5)):

(5) $$E=\frac{K_{in}}{F}\cdot 100$$

where F (µL s^−1^ g^−1^) is the perfusion flow measured with [^3^H]-diazepam (brain: 42.3 µL s^−1^ g^−1^) in rats and mice [22,25].

### 2.11. Statistical Analysis

Data were analyzed with GraphPad Prism^®^ 6.0 software (San Diego, CA, USA). The results are expressed as mean ± SD. A one-way ANOVA with Tukey’s multiple comparisons test was used when performing the relative quantification and comparison of gene expression by qRT-PCR. Unpaired t-test was used when assessing the effect of an inhibitor or age difference (Figures 4 and 5). Statistical significance was set at *p* < 0.05 for all the tests.

## 3. Results

### 3.1. Expression of Oct1-3, Mate-1 and Mdr1a Transporter Genes in Brain Microvessels of Swiss, FVB and C57BL/6 Mice

**Figure 1:**
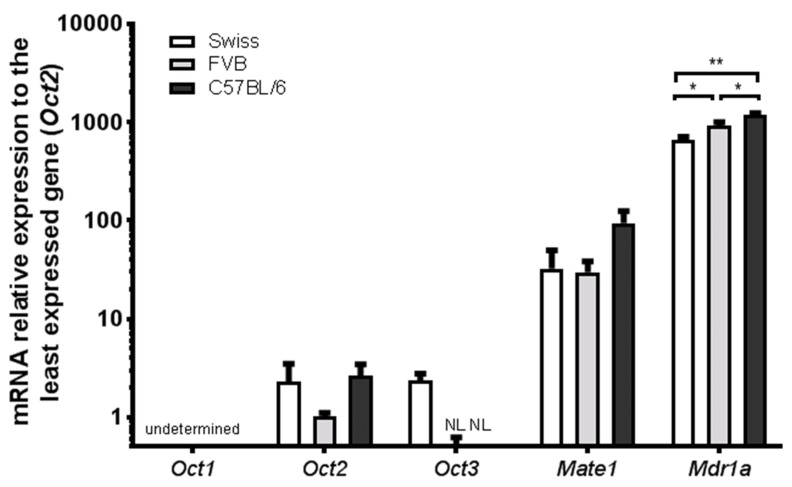
Relative expression of *Oct1*, *Oct2*, *Oct3*, *Mate1* and *Mdr1a* mRNAs in brain microvessels of Swiss, FVB, C57BL/6 mice. Relative expressions were calculated using the 2^−ΔΔCT^ method. ΔΔCT values were obtained by subtracting ΔCT values of each target gene from that of the least expressed gene (*Oct2* in FVB mice), being ΔCT the relative mRNA level of the target gene normalized to that of *β-actin*. Data are presented as mean ± S.D (*n*=3, performed in duplicate). One-way ANOVA with Tukey’s multiple comparisons test (NL: negligible levels, * *p* < 0.05, ** *p* < 0.01).

The mRNA levels of five transporters (*Oct1-3*, *Mate1*, *Mdr1a*) were analyzed and compared in the Swiss, FVB and C57BL/6 mice strains by qRT-PCR (*n* = 3, 5 mice per *n*). The *Oct1* mRNA contents were below the limit of detection (CT = undetermined), suggesting that this SLC transporter is not expressed at the BBB regardless of the mice strain. *Oct2* mRNA levels were detected in the brain microvasculature of the three strains (Figure 1), with no statistical difference between the three strains observed. Nevertheless, the expression of *Oct2* in mice brain microvessels is considerably low, as the average CT found was 32 (limit of quantification set as CT = 33). *Oct3* expression in the FVB and C57BL/6 strains is negligible (CT > 34). In Swiss mice *Oct3* expression was detected. We have previously shown that Oct3 immunostaining was obvious in nerve fibers but undetectable in cerebral vessels, either luminally or abluminally in Swiss mice [8].

On the other hand, the expression of the *Mate1* transporter gene is shown to be higher than that of *Octs* at the mice BBB (Figure 1). Still, only the expression of *Mate1* in C57BL/6 mice is significantly higher than those of other SLC transporters studied in every mouse strain (*p* < 0.05). The mRNA levels of the ABC transporter *Mdr1a* are extremely enriched in the brain microvessels of every mouse strain, evidencing a high degree of purity of each microvessel fraction. Mdr1a mRNA levels are significantly higher than any SLC transporter analyzed (*p* < 0.0001), independently of the strain, and shown to be higher in C57BL/6 than in FVB (1.3-fold, *p* < 0.05) and Swiss mice (1.8-fold, *p* < 0.01).

### 3.2. Expression of Oct1-3 Transporter Genes in Rat Brain Microvessels

**Figure 2:**
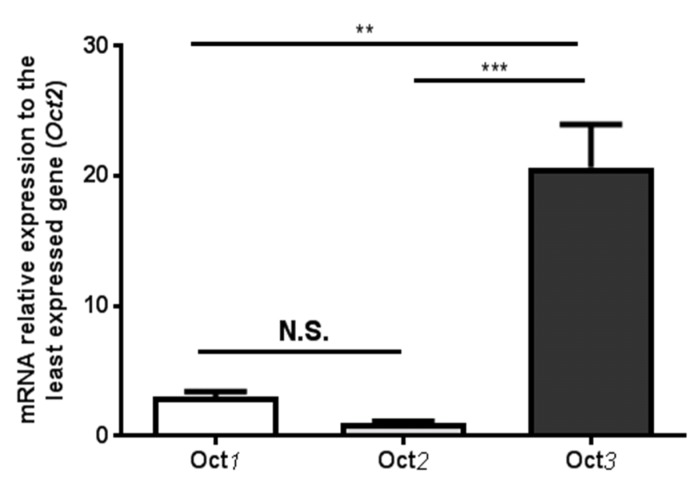
Relative expression of *Oct1*, *Oct2* and *Oct3* mRNAs in brain cortical microvessels of Sprague-Dawley rats. Relative expressions were calculated using the 2^−ΔΔCT^ method. ΔΔCT values were obtained by subtracting ΔCT values of each target gene from that of the least expressed gene (*Oct2*) in a given sample, being ΔCT the relative mRNA level of the target gene normalized to that of *β-actin*. Data are presented as mean ± S.D (n=3, performed in duplicate). One-way ANOVA with Tukey’s multiple comparisons test (NS: no statistical significance, ** *p* < 0.01, *** *p* < 0.001).

Given the low mRNA expression observed for any of the OCT transporters at the mouse BBB, we investigated the mRNA levels of these OCTs in the rat brain microvessels. As in mice, the expression of *Oct1* and *Oct2* in rat brain microvessels is negligible, given that the average CT (CT = 31 and 32, respectively; data not shown) is close to its limit of quantification. However, isolated rat brain microvessels evidenced the presence of measurable *Oct3* levels (Figure 2). *Oct3* is thus the most abundant OCT detected in rat brain microvessel samples, whose mRNA levels were 6.8-fold those of *Oct1* (*p* < 0.01) and 20.5-fold those of *Oct2* (*p* < 0.001).

### 3.3. MRM Quantification of OCT1, OCT2 and MATE1 at the Human BBB: Freshly Isolated Brain Microvessels and BMVECs

**Figure 3:**
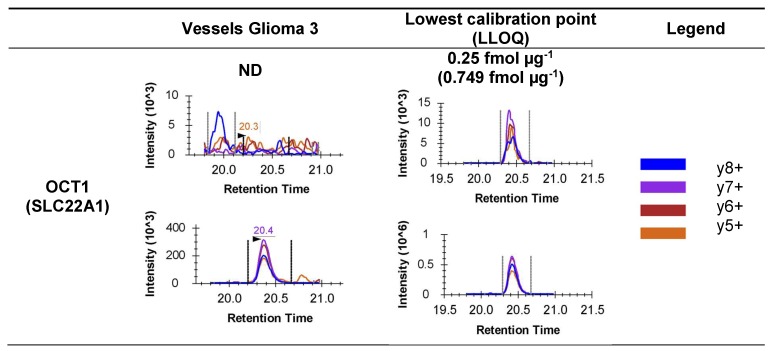

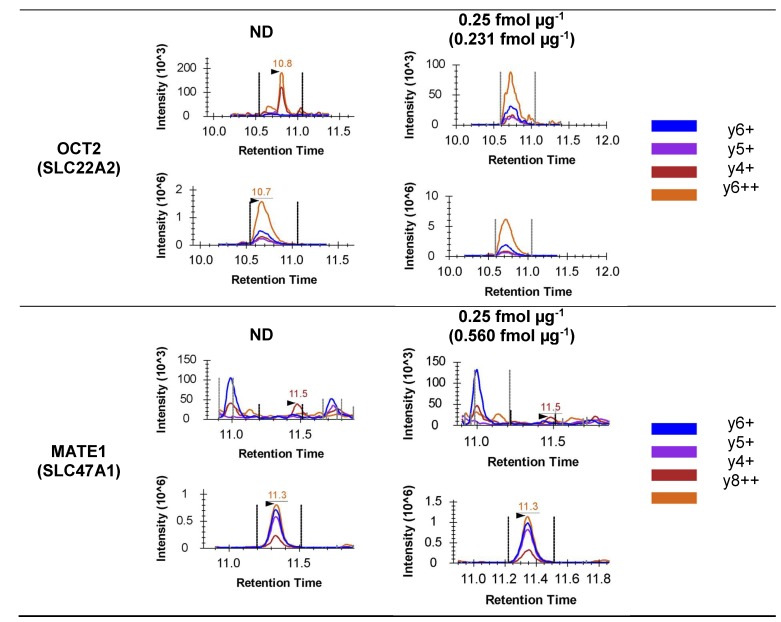
Representative chromatogram examples of the three quantified transporters OCT1 (SLC22A1), OCT2 (SLC22A2) and MATE1 (SLC47A1). The left column entitled “Vessels glioma3” shows the chromatograms obtained after analysis of the sample “Vessels glioma3”, for each of the proteins. In each box of this column, the top chromatogram is the one obtained after detection of the unlabeled peptide specific for the protein; the chromatogram at the bottom is the one obtained after detection of the labeled peptide. The absence of unlabeled peptide (therefore protein ND; not detected) is proven when there is no peak on the top chromatogram at the same retention time as the peak of the bottom chromatogram (case of OCT1, MATE1). For OCT2, the absence of the unlabeled peptide is proven because the peak at 10.8 min (top chromatogram) is the one obtained for a molecule which does not have the same relative intensity of the daughter ions (y6^+^, y5^+^, y4^+^, y6^++^) as the one in the bottom chromatogram (labeled peptide). The column “Lowest calibration point” (low limit of quantification, LLOQ) shows chromatograms obtained for each proteotypic peptide of OCT1, OCT2 and MATE1, at two different concentrations; in each box 0.25 fmol µg^−1^ (top chromatogram) and the lowest point of the calibration curve (the nearest of the LLOQ) (bottom chromatogram). The “Legend” column shows the colors used to identify the daughter ions in the chromatograms. The relative intensity of these ions must be the same for the labeled peptide and the unlabeled peptide (if detected) to prove the presence of the proteotypic peptide (and therefore of the protein).

Representative chromatograms are shown in Figure 3 for glioma vessels 3 and NP vessels 1 and the 0.25 fmol µg^−1^ point of the calibration curve, the nearest to the LLOQ. No chromatographic peak (with 3 or 4 co-eluting transitions and a fragmentation profile similar to the heavy peptide) was observed for the native peptides of the sequences analyzed for OCT1, OCT2 and MATE1 in freshly isolated human microvessels (*n* = 5) or BMVECs at P0 (*n* = 3) and P1 (*n* = 4). Therefore, SLC transporters OCT1, OCT2 and MATE1 are below the LLOQ in any of the analyzed samples (see Table 2). An example of the results of the quantification obtained in one of the biological samples are shown in Figure 3.

### 3.4. Phenylalanine and MPP^+^ Transport Studies in Primary Cultured Human BMVECs

**Figure 4:**
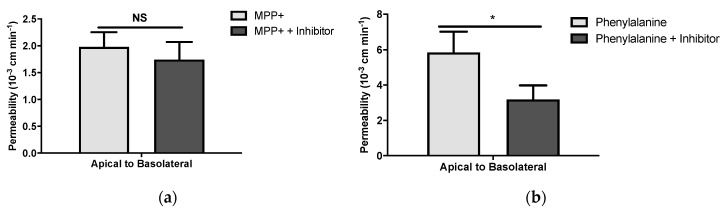
Permeability (10^−3^ cm min^−1^) from apical to basolateral passage of (**a**) [^3^H]-MPP^+^ ± tetraethylammonium (TEA) 2 mM or (**b**) of [^3^H]-Phenylalanine ± Inhibitor (Tryptophan 5 mM) on a human blood-brain barrier (BBB) model, corrected to that of [^14^C]-sucrose transport (PE; 10^−3^ cm min^−1^). Results are expressed as mean ± S.D. (*n* = 3–4 independent experiments, performed in triplicate). NS: no statistical significance, *p < 0.05 in the presence vs lack of the adequate inhibitor.

We then assessed whether OCT1-3, MATE transporters were functional in BMVECs by measuring the uptake of [^3^H]-MPP^+^ in the presence and absence of an inhibitor: TEA. Then, [^3^H]-MPP^+^ transport was investigated from the apical to the basolateral compartment, in the presence of this inhibitor. As shown in Figure 4a, TEA did not produce any significant decrease in [^3^H]-MPP^+^ uptake, suggesting the lack of function of OCT1-3 or MATE transporters. [^3^H]-L-phenylalanine uptake by SLC7A5 was measured as a control experiment, evidencing the presence of active uptake transport in human BMVECs using the same technique. The uptake of [^3^H]-L-phenylalanine was performed in the presence or absence of tryptophan, an inhibitor of SLC7A5. A significant decrease of the [^3^H]-L-phenylalanine uptake from the apical to the basolateral compartment was observed in the presence of the SLC7A5 inhibitor (Figure 4b, *n* = 3, *p* < 0.05). The cell monolayer cultivated on inserts registered a TEER of 720 ± 265 Ω cm^2^ (*n* = 7), while sucrose paracellular permeability was as low as 0.15 ± 0.07 10^−3^ cm min^−1^ (*n* = 6), thus supporting the current cell culture as a valid BBB model.

### 3.5. [^3^H]-1-Methyl-4-Phenylpyridinium Transport at the Luminal BBB in Mice and Rats

**Figure 5:**
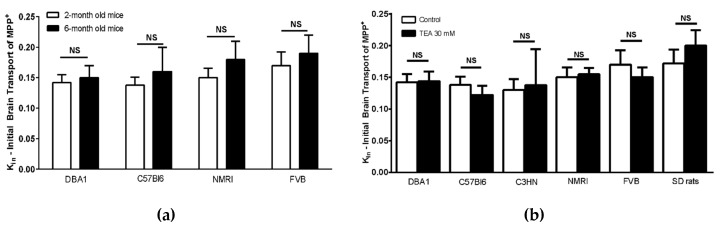
(**a**) Brain transport (Kin; μL s^−1^ g^−1^) of [^3^H]-MPP^+^ in 2-month and 6-month-old mice (DBA1, C57Bl/6, NMRI, and FVB) and (**b**) brain transport of [^3^H]-MPP^+^ with and without co-perfusion of the OCTs inhibitor TEA (30 mM), measured by in situ brain perfusion in 2-month-old mice of different strains and in Sprague Dawley (SD) rats. Results are expressed as mean ± S.D. (*n* = 5 animals per group). * *p* < 0.05 as compared with the appropriate control group. NS: no statistical significance

Low molecular weight [^14^C]-sucrose was used as a marker of the BBB integrity once it does not significantly cross plasma membranes and its paracellular diffusion is hampered by existing tight junctions. The brain transport clearance (K_in_) of [^3^H]-MPP^+^ measured in 2-month old mice and rats ranges from 0.12 to 0.20 µL s^−1^ g^−1^, which represents a luminal BBB extraction from 0.3% to 0.5%, in comparison to diazepam BBB transport (data not shown), a vascular flow marker. Differences in basal [^3^H]-MPP^+^ brain transport were not evidenced between the selected mouse strains, nor were they altered between 2-month and 6-month old NMRI, FVB, C57BL/6 and DBA1 mice (Figure 5a). This low basal BBB [^3^H]-MPP^+^ brain extraction/clearance remained unaltered when animals were co-perfused with the wide range organic cation inhibitor TEA (30 mM), for all the rat and mouse strains tested (Figure 5b). These results demonstrate that TEA sensitive carrier-mediated systems are not involved further supporting their lack of functional effect at the luminal BBB interface in the selected mouse and rat strains.

## 4. Discussion

In recent years, the presence and function of influx transporters at the BBB have been subject to analysis, notably that of transporters belonging to the SLC22 family. The polyspecific OCTs belonging to the SLC22 family are known to mediate the transport of a large number of structurally diverse organic cations, with an extensive overlap of substrates. While OCT1 and OCT2 are essentially found in the basolateral membrane of hepatocytes, enterocytes, and renal proximal tubular cells, contributing to the body clearance of xenobiotics [26], OCT3 has a more widespread distribution, including in neurons and astrocytes from different brain regions like the cerebellum, hippocampus, and cerebral cortex [27,28,29]. Here, OCT3 participates in the extraneuronal clearance of biogenic amine neurotransmitters, such as dopamine, epinephrine, norepinephrine, histamine and serotonin, are essential for a well-regulated synaptic transmission. Nonetheless, molecular and functional studies were performed either in vitro or in vivo, and using different species and strains, they have produced conflicting data concerning the presence of OCTs at the BBB.

A few *in vitro* studies performed in rat brain endothelial cell lines, such as RBE4 or TR-BBB13, evidence the lack of or negligible expression of Oct1, Oct2 and Oct3 [30,31], while Oct1 expression has been claimed to be present in rat ARBEC cells [12,32], and in the human brain microvascular endothelial cell line hCMEC/D3, expressing mRNA and protein OCT1 and OCT3, but not OCT2 [33,34]. Nonetheless, it is known that the isolation and culture of endothelial cells for use as an *in vitro* BBB model often leads to the dysregulation of transporters [35], and therefore more sophisticated models are needed to clarify such controversial results.

In the present report, the expression of OCTs at the rodent and human BBB were both explored with in vivo and in vitro, based on primary cultures studies. Our results on the expression of OCTs at the rodent BBB, obtained by qRT-PCR, reveal that independently of the mouse strain, *Oct1* is absolutely absent, and *Oct2* and *Oct3* mRNA levels are negligible. Similarly, rat brain microvessels evidence negligible levels of Oct1 and Oct2, only evidencing quantifiable *Oct3* mRNA levels. Nonetheless, given the relative abundance of Oct3 in the brain parenchyma, the small mRNA levels encoding for *Oct3* detected in rat and Swiss mice microvessels, are likely due to the contaminating non-endothelial cells remaining after the isolation of brain microvessels as no function was measured according to [^3^H]-MPP^+^ BBB transport results, nor an Oct3 immunostaining was found at the Swiss mice BBB according to our previous findings [8]. Furthermore, we evidenced by LC-MS/MS that SLC transporters OCT1, OCT2 and MATE1 protein levels are below the limit of quantification in all of the human brain microvessel samples. This thus suggests that OCT1, OCT2 and MATE1 are not present at the human BBB, or if so, in very low levels. The present results are corroborated by previous findings, where OCT1-3, PMAT and MATE1 mRNA and protein contents were also undetected or very low in Swiss mice and human isolated brain microvessels [8,10,11]. Controversially, a few studies claimed the presence of OCTs at the BBB, but such result discrepancies may possibly lie in the methodologies used in the brain microvessel purification and analysis. OCT1 and OCT2 were shown to be expressed at the luminal side of human, C57BL/6 mice and rat brain endothelial cells [12], but this study did not perform any assays to assess the degree of contamination from other brain cell types, and therefore it is not possible to exclude that such expression may derive from neuronal or glial origin. We cannot exclude that OCT1 and OCT2 immunostaining found in brain microvessels from C57BL/6 mice [9] may derive from astrocyte or pericyte contamination, or from unspecific binding of the antibodies, given that the applied procedure involving sample air dry is prone to more antigen reactivity. Protein expression of OCT3 and MATE1, but not that of OCT1 and OCT2, was also demonstrated in isolated human brain microvessels [36]. However, the brain microvessel fraction obtained has not been thoroughly enriched (2-fold reduction of neuronal and astrocytic markers vs total brain cortex only) whereas the brain microvessel enrichment using our isolation methodology accounts for a 31-fold neuronal and 7-fold astrocytic contamination reduction, in comparison to total brain cortex [10,37]. Therefore, the expression of OCT3 and MATE1 detected study may possibly come from neuronal, glial and mural cells contamination, and not necessarily evidence their presence at the human brain endothelium. According to a vascular single-cell transcriptomics study, MATE1 and OCT3 are mainly or exclusively detected in perivascular fibroblast-like cells, that are present in all brain vessel types, except brain capillaries [38]. The mechanical isolation used in our study allows one to obtain the enriched brain capillaries fraction, but could not exclude larger brain vessels contamination and the presence of perivascular matrix associated cells.

The functional studies that we have carried out in different models further confirm the non-involvement of [^3^H]-MPP^+^ transporters on the luminal side of the BBB, whereas this method and substrate has proven its ability to measure the function of OCTs at the blood-retinal barriers [8]. The results obtained by the in situ brain perfusion of different mouse strains and rats on the brain transport clearance (K_in_) of [^3^H]-MPP^+^, a broad substrate of OCT1-3 and MATE1, evidence that its BBB permeability is not carrier-mediated, since the co-perfusion with the wide range OCT inhibitor TEA did not alter the luminal BBB permeability to [^3^H]-MPP^+^. A considerable low luminal BBB permeability to [^3^H]-MPP^+^ (0.12 to 0.20 µL s^−1^ g^−1^) was found, which may result from poor passive diffusion, and which did not differ between the different mouse strains, nor between rats and mice. Additionally, we were not able to see any [^3^H]-MPP^+^ transport modulation in the presence of TEA in our human BBB model in vitro, suggesting a lack of functional activity for OCTs and MATE1 at the human BBB. These results are in concordance with previous findings evidencing no modification of the [^3^H]-MPP^+^ brain transport in FVB (wild-type, *Oct1/2*^(−/−)^, and *Oct3*^(−/−)^) and in Swiss mice (+/- TEA) [8], nor of [^14^C]-TEA brain transport in FVB *Oct1*^(−/−)^mice [39], further confirming that these transporters are not functional at the BBB. As most of the human samples were from pathological BBB, we cannot exclude that the lack of expression could be artefactual. Nonetheless, based on two non-pathological samples obtained from patients this trend was also confirmed. In contrast with our results, functional presence of OCT1 and/or OCT2 at the BBB has been suggested in vitro [12,32,33], with the above mentioned limitations, but also in some specific mice strains (i.e., C57/Bl6) with age dependent Oct1/2 BBB activity [9,12,32]. However, these in vivo results were based on brain ISF microdialysis sampling which, unlike our in vivo methodology, cannot discriminate whether the measured concentrations result from kinetic processes that occurred at the BBB or at other brain ISF interfaces, such as neurons, glial cells or ventricles, where OCT2-3 transporters may be present [40,41].

The lack of a carrier-mediated transport for biogenic amines (e.g., dopamine, histamine) or xenobiotic cations, such as MPP^+^, aflatoxin B1, and paraquat, at the BBB, as evidenced in the present study, leads us to conclude that the BBB plays its role of limiting their delivery into the CNS. Many of the xenobiotic compounds transported by OCTs are neurotoxicants, and therefore the lack of such transporters at the BBB are coherent with the BBB function of preventing neurotoxins, either endogenous metabolites or environmental xenobiotics in the blood circulation from entering the brain. It is well recognized that paraquat, an OCT substrate, has a limited neurotoxicity due to its limited brain uptake: it is weakly sequestered in specific brain structures, namely the pineal gland and the lateral ventricles, which either lie outside the BBB or do not have a BBB [42,43,44,45]. Additionally, different mammals have demonstrated that they are relatively resistant to MPTP neurotoxicity if administered systemically [46,47], but not if infused directly into the brain, or via a nasal cavity, which exhibits a weak BBB [48]. Species differences regarding the neurotoxicity of MPTP have been described as a result of variations in the ability of MPTP to reach the CNS [49]. Given that MPTP is uncharged and more lipophilic, and consequently more prone to penetrate into the brain than its polar and charged MPP^+^ metabolite, such variations are likely due to interspecies differences in its metabolism to MPP^+^ by peripheric monoamine oxidase: the higher the activity of peripheric monoamine oxidase, the greater the MPP^+^ systemic levels are, and thus less able to reach the brain parenchyma to exert its neurotoxicity [50]. Knowing that the concentration/extent of a compound within the ISF, although primarily controlled by the BBB, is also dependent of the transport processes present at the level of glial and neuronal cell membranes, as known at the synaptic cleft, the presence of OCTs on these cells appears to more likely support the pharmacokinetic-pharmacodynamic interspecies variability for OCT substrates for the brain tissue. Indeed, our results support that the absence of OCTs/MATE1 at the BBB helps in the prevention of the brain uptake of their substrates like MPP^+^ or paraquat, even though paraquat may be able to reach the CNS via LAT1 (SLC7A5) BBB transport [4,5], it is also extruded from the CNS wherever a BBB lies, probably via P-gp, since paraquat is also a recognized substrate of this ABC efflux transporter [51,52,53].

## 5. Conclusions

In conclusion, the results of the present study evidence a lack of expression of OCT1 and OCT2 in either mice, rat or human brain microvessels, and even though an expression of Oct3 and Mate1 is detected in mice brain microvessels, MATE1 expression was not verified at the human BBB. Functional studies conducted in models representative of the three species further evidenced an absence of active OCTs carriers at the BBB. Therefore, the role of BBB in maintaining ISF homeostasis in the CNS does not appear to be dependent on OCTs, thus limiting the systemic strategies of drug delivery to the brain targeting OCT transporters.

## Figures and Tables

**Table 1:** Primer Sequences Used for SYBR Green-Based quantitative Real Time-Polymerase Chain Reaction (qRT-PCR).

Gene	Forward Primer (Sense) (5′–3′)	Reverse Primer (Antisense) (5′–3′)	Length (bp)
β*-actin*	CTGGCCCGGACCTGACAGA	GCGGCAGTGGCCATCTCTC	132
*Oct-1 mice*	ATAGCGGCATCAAATCTGGT	GACAAGCGAGGGTCACATTC	94
*Oct-2 mice*	GAGCCACTTGCCACTGAG	ATCCAGGAGTGAGCACACACTAG	102
*Oct-3 mice*	GTGAGCCAGTTTGACCTTGTCT	GATGAGCCTGCCATATCTGTC	129
*Mate-1 mice*	GCCATCGTTAATGCCATCGGGTA	CAGGCCGATCACTCCCAGCTT	90
*Oct-1 rat*	CGGTGGCTGTTGTCCCAGA	GAGCATCTTCAGGTCAGCAGG	102
*Oct-2 rat*	GCCGGTCTCTCTTCAGAACCT	TGTGTTTCCTTATCTGAGGGGT	101
*Oct-3 rat*	CAGCCAGTTTGACCTTGTCT	GTAAAAGCCCCAGCCAGGA	90

**Table 2:** Calibration curve parameters. Definitions: Limit of detection (LOD), lower limit of quantification (LLOQ), upper limit of quantification (ULOQ).

		fmol µg^−1^					
Protein	Peptide	LOD	LLOQ	ULOQ	Slope	Intercept	R²
SLC22A1	LSPSFADLFR	0.48	0.75	62.5	1.243	−0.110	0.9855
SLC22A2	SLPASLQR	0.15	0.23	125	1.037	−0.018	0.9996
SLC47A1	GGPEATLEVR	0.35	0.56	62.5	1.794	0.339	0.9984

## References

[1] Chaves C., Remiao F., Cisternino S., Decleves X. (2017). Opioids and the blood-brain barrier: A dynamic interaction with consequences on drug disposition in brain. Curr. Neuropharmacol..

[2] Pardridge W.M., Oldendorf W.H. (1975). Kinetics of blood-brain transport of hexoses. Biochim. Biophys. Acta.

[3] Pardridge W.M., Boado R.J., Farrell C.R. (1990). Brain-type glucose transporter (GLUT-1) is selectively localized to the blood-brain barrier. Studies with quantitative western blotting and in situ hybridization. J. Biol. Chem..

[4] Pardridge W.M., Oldendorf W.H. (1975). Kinetic analysis of blood-brain barrier transport of amino acids. Biochim. Biophys. Acta.

[5] Boado R.J., Li J.Y., Nagaya M., Zhang C., Pardridge W.M. (1999). Selective expression of the large neutral amino acid transporter at the blood-brain barrier. Proc. Natl. Acad. Sci. USA.

[6] Stoll J., Wadhwani K.C., Smith Q.R. (1993). Identification of the cationic amino acid transporter (System y+) of the rat blood-brain barrier. J. Neurochem..

[7] Lozano E., Herraez E., Briz O., Robledo V.S., Hernandez-Iglesias J., Gonzalez-Hernandez A., Marin J.J. (2013). Role of the plasma membrane transporter of organic cations OCT1 and its genetic variants in modern liver pharmacology. Biomed. Res. Int..

[8] Andre P., Saubamea B., Cochois-Guegan V., Marie-Claire C., Cattelotte J., Smirnova M., Schinkel A.H., Scherrmann J.M., Cisternino S. (2012). Transport of biogenic amine neurotransmitters at the mouse blood-retina and blood-brain barriers by uptake1 and uptake2. J. Cereb. Blood Flow Metab..

[9] Wu K.C., Lu Y.H., Peng Y.H., Tsai T.F., Kao Y.H., Yang H.T., Lin C.J. (2015). Decreased expression of organic cation transporters, Oct1 and Oct2, in brain microvessels and its implication to MPTP-induced dopaminergic toxicity in aged mice. J. Cereb. Blood Flow Metab..

[10] Uchida Y., Ohtsuki S., Katsukura Y., Ikeda C., Suzuki T., Kamiie J., Terasaki T. (2011). Quantitative targeted absolute proteomics of human blood-brain barrier transporters and receptors. J. Neurochem..

[11] Shawahna R., Uchida Y., Decleves X., Ohtsuki S., Yousif S., Dauchy S., Jacob A., Chassoux F., Daumas-Duport C., Couraud P.O. (2011). Transcriptomic and quantitative proteomic analysis of transporters and drug metabolizing enzymes in freshly isolated human brain microvessels. Mol. Pharm..

[12] Lin C.J., Tai Y., Huang M.T., Tsai Y.F., Hsu H.J., Tzen K.Y., Liou H.H. (2010). Cellular localization of the organic cation transporters, OCT1 and OCT2, in brain microvessel endothelial cells and its implication for MPTP transport across the blood-brain barrier and MPTP-induced dopaminergic toxicity in rodents. J. Neurochem..

[13] Yousif S., Saubamea B., Cisternino S., Marie-Claire C., Dauchy S., Scherrmann J.M., Decleves X. (2008). Effect of chronic exposure to morphine on the rat blood-brain barrier: Focus on the P-glycoprotein. J. Neurochem..

[14] Perriere N., Demeuse P., Garcia E., Regina A., Debray M., Andreux J.P., Couvreur P., Scherrmann J.M., Temsamani J., Couraud P.O. (2005). Puromycin-based purification of rat brain capillary endothelial cell cultures. Effect on the expression of blood-brain barrier-specific properties. J. Neurochem..

[15] Chaves C., Gomez-Zepeda D., Auvity S., Menet M.C., Crété D., Labat L., Remiao F., Cisternino S., Decleves X. (2016). Effect of subchronic intravenous morphine infusion and naloxone-precipitated morphine withdrawal on P-gp and Bcrp at the rat blood-brain barrier. J. Pharm. Sci..

[16] Gerber S.A., Rush J., Stemman O., Kirschner M.W., Gygi S.P. (2003). Absolute quantification of proteins and phosphoproteins from cell lysates by tandem MS. Proc. Natl. Acad. Sci. USA.

[17] Kamiie J., Ohtsuki S., Iwase R., Ohmine K., Katsukura Y., Yanai K., Sekine Y., Uchida Y., Ito S., Terasaki T. (2008). Quantitative atlas of membrane transporter proteins: Development and application of a highly sensitive simultaneous LC/MS/MS method combined with novel in-silico peptide selection criteria. Pharm. Res..

[18] Mani D.R., Abbatiello S.E., Carr S.A. (2012). Statistical characterization of multiple-reaction monitoring mass spectrometry (MRM-MS) assays for quantitative proteomics. BMC Bioinf..

[19] Pelkonen L., Sato K., Reinisalo M., Kidron H., Tachikawa M., Watanabe M., Uchida Y., Urtti A., Terasaki T. (2017). LC-MS/MS Based Quantitation of ABC and SLC Transporter Proteins in Plasma Membranes of Cultured Primary Human Retinal Pigment Epithelium Cells and Immortalized ARPE19 Cell Line. Mol. Pharm..

[20] Maclean B., Tomazela D.M., Abbatiello S.E., Zhang S., Whiteaker J.R., Paulovich A.G., Carr S.A., Maccoss M.J. (2010). Effect of collision energy optimization on the measurement of peptides by selected reaction monitoring (SRM) mass spectrometry. Anal. Chem..

[21] MacLean B., Tomazela D.M., Shulman N., Chambers M., Finney G.L., Frewen B., Kern R., Tabb D.L., Liebler D.C., MacCoss M.J. (2010). Skyline: An open source document editor for creating and analyzing targeted proteomics experiments. Bioinformatics.

[22] Cattelotte J., Andre P., Ouellet M., Bourasset F., Scherrmann J.M., Cisternino S. (2008). In situ mouse carotid perfusion model: Glucose and cholesterol transport in the eye and brain. J. Cereb. Blood Flow Metab..

[23] Cisternino S., Rousselle C., Debray M., Scherrmann J.M. (2003). In vivo saturation of the transport of vinblastine and colchicine by P-glycoprotein at the rat blood-brain barrier. Pharm. Res..

[24] Takasato Y., Rapoport S.I., Smith Q.R. (1984). An in situ brain perfusion technique to study cerebrovascular transport in the rat. Am. J. Physiol..

[25] Dagenais C., Rousselle C., Pollack G.M., Scherrmann J.M. (2000). Development of an in situ mouse brain perfusion model and its application to mdr1a P-glycoprotein-deficient mice. J. Cereb. Blood Flow Metab..

[26] Jonker J.W., Schinkel A.H. (2004). Pharmacological and physiological functions of the polyspecific organic cation transporters: OCT1, 2, and 3 (SLC22A1-3). J. Pharmacol. Exp. Ther..

[27] Grundemann D., Schechinger B., Rappold G.A., Schomig E. (1998). Molecular identification of the corticosterone-sensitive extraneuronal catecholamine transporter. Nat. Neurosci..

[28] Inazu M., Takeda H., Matsumiya T. (2003). Expression and functional characterization of the extraneuronal monoamine transporter in normal human astrocytes. J. Neurochem..

[29] Wu X., Kekuda R., Huang W., Fei Y.J., Leibach F.H., Chen J., Conway S.J., Ganapathy V. (1998). Identity of the organic cation transporter OCT3 as the extraneuronal monoamine transporter (uptake2) and evidence for the expression of the transporter in the brain. J. Biol. Chem..

[30] Friedrich A., George R.L., Bridges C.C., Prasad P.D., Ganapathy V. (2001). Transport of choline and its relationship to the expression of the organic cation transporters in a rat brain microvessel endothelial cell line (RBE4). Biochim. Biophys. Acta.

[31] Okura T., Kato S., Takano Y., Sato T., Yamashita A., Morimoto R., Ohtsuki S., Terasaki T., Deguchi Y. (2011). Functional characterization of rat plasma membrane monoamine transporter in the blood-brain and blood-cerebrospinal fluid barriers. J. Pharm. Sci..

[32] Liou H.H., Hsu H.J., Tsai Y.F., Shih C.Y., Chang Y.C., Lin C.J. (2007). Interaction between nicotine and MPTP/MPP+ in rat brain endothelial cells. Life Sci..

[33] Dickens D., Owen A., Alfirevic A., Giannoudis A., Davies A., Weksler B., Romero I.A., Couraud P.O., Pirmohamed M. (2012). Lamotrigine is a substrate for OCT1 in brain endothelial cells. Biochem. Pharmacol..

[34] Dos Santos Pereira J.N., Tadjerpisheh S., Abu Abed M., Saadatmand A.R., Weksler B., Romero I.A., Couraud P.O., Brockmoller J., Tzvetkov M.V. (2014). The poorly membrane permeable antipsychotic drugs amisulpride and sulpiride are substrates of the organic cation transporters from the SLC22 family. AAPS J..

[35] Lyck R., Ruderisch N., Moll A.G., Steiner O., Cohen C.D., Engelhardt B., Makrides V., Verrey F. (2009). Culture-induced changes in blood-brain barrier transcriptome: Implications for amino-acid transporters in vivo. J. Cereb. Blood Flow Metab..

[36] Geier E.G., Chen E.C., Webb A., Papp A.C., Yee S.W., Sadee W., Giacomini K.M. (2013). Profiling solute carrier transporters in the human blood-brain barrier. Clin. Pharmacol. Ther..

[37] Yousif S., Marie-Claire C., Roux F., Scherrmann J.M., Decleves X. (2007). Expression of drug transporters at the blood-brain barrier using an optimized isolated rat brain microvessel strategy. Brain Res..

[38] Vanlandewijck M., He L., Mäe M.A., Andrae J., Ando K., Del Gaudio F., Nahar K., Lebouvier T., Laviña B., Gouveia L. (2018). Molecular Atlas of Cell Types and Zonation in the Brain Vasculature. Nature.

[39] Jonker J.W., Wagenaar E., Mol C.A., Buitelaar M., Koepsell H., Smit J.W., Schinkel A.H. (2001). Reduced hepatic uptake and intestinal excretion of organic cations in mice with a targeted disruption of the organic cation transporter 1 (Oct1 [Slc22a1]) gene. Mol. Cell. Biol..

[40] Amphoux A., Vialou V., Drescher E., Bruss M., Mannoury La Cour C., Rochat C., Millan M.J., Giros B., Bonisch H., Gautron S. (2006). Differential pharmacological in vitro properties of organic cation transporters and regional distribution in rat brain. Neuropharmacology.

[41] Vialou V., Amphoux A., Zwart R., Giros B., Gautron S. (2004). Organic cation transporter 3 (Slc22a3) is implicated in salt-intake regulation. J. Cereb. Blood Flow Metab..

[42] Bartlett R.M., Holden J.E., Nickles R.J., Murali D., Barbee D.L., Barnhart T.E., Christian B.T., DeJesus O.T. (2009). Paraquat is excluded by the blood brain barrier in rhesus macaque: An in vivo pet study. Brain Res..

[43] Naylor J.L., Widdowson P.S., Simpson M.G., Farnworth M., Ellis M.K., Lock E.A. (1995). Further evidence that the blood/brain barrier impedes paraquat entry into the brain. Hum. Exp. Toxicol..

[44] Widdowson P.S., Farnworth M.J., Upton R., Simpson M.G. (1996). No changes in behaviour, nigro-striatal system neurochemistry or neuronal cell death following toxic multiple oral paraquat administration to rats. Hum. Exp. Toxicol..

[45] Bartlett R.M., Murali D., Nickles R.J., Barnhart T.E., Holden J.E., DeJesus O.T. (2011). Assessment of fetal brain uptake of paraquat in utero using in vivo PET/CT imaging. Toxicol. Sci..

[46] Chiueh C.C., Markey S.P., Burns R.S., Johannessen J.N., Jacobowitz D.M., Kopin I.J. (1984). Neurochemical and behavioral effects of 1-methyl-4-phenyl-1,2,3,6- tetrahydropyridine (MPTP) in rat, guinea pig, and monkey. Psychopharmacol. Bull..

[47] Boyce S., Kelly E., Reavill C., Jenner P., Marsden C.D. (1984). Repeated administration of N-methyl-4-phenyl 1,2,5,6-tetrahydropyridine to rats is not toxic to striatal dopamine neurones. Biochem. Pharmacol..

[48] Rojo A.I., Montero C., Salazar M., Close R.M., Fernandez-Ruiz J., Sanchez-Gonzalez M.A., de Sagarra M.R., Jackson-Lewis V., Cavada C., Cuadrado A. (2006). Persistent penetration of MPTP through the nasal route induces Parkinson’s disease in mice. Eur. J. Neurosci..

[49] Kalaria R.N., Mitchell M.J., Harik S.I. (1987). Correlation of 1-methyl-4-phenyl-1,2,3,6-tetrahydropyridine neurotoxicity with blood-brain barrier monoamine oxidase activity. Proc. Natl. Acad. Sci. USA.

[50] Kalaria R.N., Harik S.I. (1987). Blood-brain barrier monoamine oxidase: Enzyme characterization in cerebral microvessels and other tissues from six mammalian species, including human. J. Neurochem..

[51] Vilas-Boas V., Silva R., Guedes-de-Pinho P., Carvalho F., Bastos M.L., Remiao F. (2014). RBE4 cells are highly resistant to paraquat-induced cytotoxicity: Studies on uptake and efflux mechanisms. J. Appl. Toxicol..

[52] Silva R., Palmeira A., Carmo H., Barbosa D.J., Gameiro M., Gomes A., Paiva A.M., Sousa E., Pinto M., de Lourdes Bastos M. (2015). P-glycoprotein induction in Caco-2 cells by newly synthetized thioxanthones prevents paraquat cytotoxicity. Arch. Toxicol..

[53] Dinis-Oliveira R.J., Remiao F., Duarte J.A., Ferreira R., Sanchez Navarro A., Bastos M.L., Carvalho F. (2006). P-glycoprotein induction: An antidotal pathway for paraquat-induced lung toxicity. Free Radic. Biol. Med..

